# HMGB1 Protects the Heart Against Ischemia–Reperfusion Injury via PI3K/AkT Pathway-Mediated Upregulation of VEGF Expression

**DOI:** 10.3389/fphys.2019.01595

**Published:** 2020-01-29

**Authors:** Yan-Hong Zhou, Qian-Feng Han, Lei Gao, Ying Sun, Zhan-Wei Tang, Meng Wang, Wei Wang, Heng-Chen Yao

**Affiliations:** ^1^Department of Cardiology, Liaocheng People’s Hospital Affiliated to Shandong University and Clinical School of Shandong First Medical University, Liaocheng, China; ^2^Zhong Yuan Academy of Biological Medicine, Liaocheng People’s Hospital, Shandong University, Liaocheng, China; ^3^Department of Cardiology, Jinan Central Hospital, Shandong University, Jinan, China

**Keywords:** myocardial ischemia–reperfusion injury, high mobility group box 1, vascular endothelial growth factor, PI3K/Akt pathway, acute myocardial infarction

## Abstract

Delivery of exogenous high mobility group box 1 (HMGB1) may exert a beneficial effect on myocardial ischemia–reperfusion (I/R) injury. Since the expression of vascular endothelial growth factor (VEGF) and phosphatidylinositol 3-kinase/protein kinase B (PI3K/Akt) in the myocardium mediates the cardioprotective function of basic fibroblast growth factor, we hypothesized that VEGF and the PI3K/Akt signaling pathway also mediate the protective effects of intravenously delivered HMGB1. Thus, the objective of the present study was to analyze the impact of intravenous administration of HMGB1 on the myocardial expression of VEGF, myocardial fibrosis, and cardiac function in rats subjected to acute myocardial I/R. The ischemia was induced by ligation of the left anterior descending coronary artery for 30 min and was followed by 3 h of reperfusion. Myocardial malondialdehyde content, infarct size, and collagen volume fraction decreased, while the activity of superoxide dismutase was increased, the expression of VEGF and p-Akt was upregulated, and cardiac function was improved in the HMGB1-treated group when compared with rats subjected to I/R only (all *P* < 0.05). However, these effects of HMGB1 were abolished by LY294002. The obtained results demonstrate that the cardioprotective effects of intravenous administration of HMGB1 prior to I/R may be mediated by upregulation of myocardial expression of VEGF, which may activate the PI3K/Akt signaling pathway.

## Introduction

Acute myocardial infarction (MI) is a leading cause of mortality and morbidity (Zweier and Talukder, 2006). When an acute MI occurs, timely restoration of blood flow to the ischemic myocardium is essential to save the myocardial tissue from ensuing necrosis. However, the reperfusion of ischemic myocardium may itself result in severe myocardial damage, an effect known as ischemia–reperfusion (I/R) injury (Yellon and Hausenloy, 2007; Eltzschig and Eckle, 2011). Myocardial I/R injury triggers several adverse cardiovascular outcomes, such as cardiac dysfunction, coronary artery spasm, cardiac arrest, and death (Frank et al., 2012). Therefore, attenuating I/R injury is critical in the management of acute MI.

Vascular endothelial growth factor (VEGF) is an angiogenic growth factor stimulating proliferation, differentiation, migration, and survival of vascular endothelial cells (Dvorak et al., 1995; Xie et al., 2004) and inhibiting their apoptosis (Gupta et al., 1999). Additionally, several studies on myocardial I/R injury documented that VEGF plays a significant role in cardioprotection. It has been demonstrated that VEGF expression is upregulated in acute myocardial ischemia, and levels of VEGF are inversely correlated with infarct size (IS) in a rat model of MI (Yao et al., 2013a). Moreover, administration of exogenous VEGF improves functional recovery of the myocardium I/R injury (Guzman et al., 2008). Therefore, modulation of the expression of VEGF566694844566694844 may reduce tissue injury following acute myocardial ischemia. Consequently, drugs increasing myocardial VEGF expression may have a therapeutic effect on I/R-related myocardial injury.

High mobility group box 1 (HMGB1) is a ubiquitous and abundant nuclear protein (Lakhan et al., 2009) with a critical role in many cardiovascular diseases, such as atherosclerosis, MI, myocardial I/R injury, and heart failure (Ding and Yang, 2010; Yao et al., 2013b, 2016; Liu et al., 2015). Specifically, inhibition of myocardial expression of HMGB1 attenuates the extent of myocardial injury in rats following myocardial I/R (Yao et al., 2015). Additionally, administration of exogenous HMGB1 decreases IS and improves myocardial function; these effects are associated with suppressed myocardial inflammation (Abarbanell et al., 2011; Zhou et al., 2012; Zhang et al., 2015; Yao et al., 2016). However, whether exogenous HMGB1 affects the myocardial expression of VEGF remains unknown.

Phosphatidylinositol 3-kinase/protein kinase B (PI3K/Akt) constitutes a family of conserved signal transduction enzymes regulating cellular inflammatory responses and apoptosis (Cantley, 2002). The PI3K/Akt signaling pathway is essential in the protection of the myocardium against I/R injury (Chen et al., 2014; Yu et al., 2014). Recently, our laboratory was the first to report that intravenously delivered HMGB1 protects the heart from I/R injury (Zhang et al., 2015) through the upregulation of hypoxia-inducible factor-1α (HIF-1α) in the myocardium. This effect can be mediated by the activation of the protein kinase B-dependent signaling pathway (Yao et al., 2016), or by the inhibition of the p38 MAPK pathway (Zhou Y.H. et al., 2017).

Myocardial I/R injury results in cardiac fibrosis, which in turn leads to cardiac dysfunction negatively affecting the outcome of MI. Therefore, the reduction of I/R injury and the promotion of the recovery of myocardial function are currently a focus of significant research effort. In previous studies, HMGB1 was administered by direct injection into the peri-infarct area in various animal models. However, little is known regarding the impact of intravenous delivery of HMGB1 on I/R injury and subsequent myocardial dysfunction. Furthermore, VEGF is the downstream effector of HIF-1α and was demonstrated to be critically involved in the cardioprotection afforded by basic fibroblast growth factor (bFGF) in a rat model of acute MI (Yao et al., 2013a). Thus, the question remains whether intravenous injection of HMGB1 can increase the expression of VEGF in the myocardium, attenuate myocardial I/R injury, and promote cardiac function in rats following acute MI. Additionally, the related underlying mechanisms are uncertain. Therefore, the goal of the present study was to assess the impact of intravenous delivery of HMGB1 on the expression of VEGF in the myocardium, myocardial I/R injury, and cardiac function in a rat model of I/R. Importantly, the mechanisms underlying these effects were also investigated.

## Materials and Methods

### Animal Groups

All animals were fed a standard diet and received humane care. The animal experiments were performed in accordance with the “Guide for the Care and Use of Laboratory Animals” published by the US National Institutes of Health (NIH No. 85-23, revised 1996), and all protocols involving animals were reviewed and approved by the Ethics Committee of Liaocheng People’s Hospital (Liaocheng, China).

Male Wistar rats (*n* = 50, body weight 250–300 g) were obtained from the experimental laboratory of Shandong Lukang Ltd., Company (Jining, China). The animals were kept at room temperature (24°C) with a 12-h light–dark cycle and were given free access to food and water.

The rats were randomly divided into 5 groups of 10 animals each: (1) sham-operated rats (sham group); (2) rats subjected to I/R (I/R group); (3) rats receiving intravenous injection of 200 ng of recombinant HMGB1 at 30 min before the I/R protocol (HMGB1 group); (4) rats pretreated intravenously with 0.3 mg/kg of LY294002, an inhibitor of phosphoinositide 3-kinase (PI3K), at 40 min before the I/R protocol (LY group); and (5) I/R rats pretreated with an intravenous injection of HMGB1 (200 ng/kg, 30 min before ischemia) and LY294002 (0.3 mg/kg, 40 min before ischemia) (HMGB1 + LY group). HMGB1 and LY were injected into the tail vein in a volume of 0.5 ml. The sham group received an intravenous injection of 0.5 ml of normal saline.

### Animal Model

The rat I/R model was generated according to the method previously used in our laboratory (Yao et al., 2016). Under general anesthesia (sodium pentobarbital, 60 mg/kg, i.p.), the trachea was cannulated for artificial ventilation with room air at the rate of 55 breaths/min. An electric heating pad was used to maintain the body temperature at 37.0 ± 0.5°C. Lead II of the electrocardiogram (ECG) was recorded and analyzed by an ECG-6511 data acquisition system (Guangdian Medical Device Co., Shanghai, China). The I/R rats were subjected to the left anterior descending coronary artery (LAD) ligation for 30 min and subsequent reperfusion for 3 h. In the sham group, the suture was placed at the origin of the LAD, but the ligation of the artery was not performed. Before the surgical procedure, rats were fasted for 12 h and only allowed free access to water.

### Measurement of the Myocardial Level of Malondialdehyde and the Activity of Superoxide Dismutase

After 3 h of reperfusion, the hearts were harvested, washed with normal saline, and frozen at −70°C for subsequent experiments. Ischemic heart tissue, 0.5 g, was ground using a liquid nitrogen-chilled tissue pulverizer at 0–4°C. The myocardial homogenate was centrifuged at 3,500 rpm for 30 min, and the supernatant was collected and stored at −80°C. Thiobarbituric acid reactive substance assay was used to determine the MDA concentration by measuring the absorbance value at a wavelength of 532 nm. The activity of SOD was assessed by the xanthine oxide method; the absorbance value was measured at a wavelength of 550 nm. The determinations were performed using the MDA Assay kit and SOD Assay kit purchased from the Nanjing Jiancheng Bioengineering Ltd. (Nanjing, China) following the manufacturer’s instructions.

### Histological Analysis of Myocardium

Hearts were harvested and fixed in 10% buffered formalin solution for 60 min at room temperature and then for 24 h at 4°C. The specimens were paraffin-embedded, cut into 5 μm thick sections and stained with hematoxylin and eosin (HE). Images were acquired using a light microscope (Nikon/80i, Japan) and a digital camera (DP71CCD, Olympus, Japan).

### Assessment of Infarct Size

Infarct size was determined by staining with 2,3,5-triphenyltetrazolium chloride (TTC) as previously performed in our laboratory (Yao et al., 2016). At the end of reperfusion, the heart was excised, washed in phosphate-buffered saline, frozen at −80°C, and cut transversely into five slices from the apex to the base. The slices were incubated in 1% TTC (pH 7.4) at 37°C for 15 min, fixed in 10% formaldehyde solution, and imaged using a digital camera. Subsequently, the area of the infarcted (white) and surviving myocardium was measured using Image-Pro Plus 3.0 (Media Cybernetics, Silver Spring, MD, United States). IS was expressed as the percentage of the total section area of left ventricle occupied by the infarct.

### Assessment of Left Ventricular Function

Four weeks after the surgery, rats were subjected to general anesthesia by intraperitoneal injection of phenobarbital (60 mg/kg) and underwent transthoracic echocardiography with an HP Sonos 5500 instrument equipped with a 10 MHz probe. Two-dimensional mode and color Doppler echocardiograms were performed to measure the left ventricular ejection fraction (LVEF), left ventricular end-diastolic diameter (LVEDD), and left ventricular fractional shortening (LVFS). Each determination was based on 10 cardiac cycles.

### Masson Staining of the Myocardium

Myocardial sections were stained with Masson’s trichrome to determine the magnitude of myocardial fibrosis. The collagen volume fraction (CVF) was calculated using the Image-Pro Plus 6.0 (Media Cybernetics, Houston, TX, United States) and expressed as the fraction of the tissue occupied by collagen.

### Western Blotting

Western blotting was performed on the infarct samples using antibodies against VEGF, p-Akt, and β-actin. Total protein was extracted from the myocardial tissue, separated by SDS-polyacrylamide agarose gel electrophoresis (SDS-PAGE), and transferred to polyvinylidene fluoride (PVDF) membranes (Bio-Rad, Hercules, CA, United States) using 50 μg of lysate per lane. Membranes were blocked with 5% non-fat milk and 0.07% Tween 20 in Tris-buffered saline (TBS) at room temperature for 60 min and subsequently incubated overnight at 4°C with primary antibodies diluted 1:3,000. The membranes were washed and incubated with a secondary antibody, IRDye680 goat antirabbit IgG (LI-COR Bioscience) diluted 1:2,000. Bound antibodies were visualized by chemiluminescence, and protein bands were detected by the ChemiDoc imaging system (Bio-Rad, Hercules, CA, United States). The expression of VEGF and p-Akt was normalized to β-actin and total Akt, respectively.

### Statistical Analysis

Data are expressed as means ± SD or percentages where appropriate. SAS 6.12 software was used for statistical calculations. One-way ANOVA was used to analyze differences of means between groups. *P* < 0.05 was considered statistically significant.

## Results

### MDA Levels and SOD Activity

As listed in Table 1, in comparison with the sham group, the content of MDA in the myocardium significantly increased, and the activity of SOD decreased in the I/R group (*P* < 0.01 and 0.05, respectively). These changes were significantly attenuated by 566694845566694845HMGB1 pretreatment (*P* < 0.05 for both). However, the reduction in MDA contents and the increase in SOD activity induced by HMGB1 were abolished almost completely in both LY and LY + HMGB1 groups when compared to the HMGB group (*P* < 0.05 for both).

**TABLE 1:** Comparison of variables in each groups.

Variables	Sham	I/R	HMGB1	LY	LY + HMGB1
SOD (μ/mg)	150.8 ± 11.26	75.43 ± 4.97^a^	120.67± 10.35^ab^	65.97 ± 3.75^ac^	65.97 ± 3.75^ac^
MDA (nmol/mg)	1.57 ± 0.26	9.17 ± 0.52^a^	4.12 ± 0.38^ab^	8.04 ± 0.41^ac^	8.94 ± 0.43^ac^
LVIDs (mm)	4.13 ± 0.78	5.67 ± 0.93^a^	4.71 ± 0.53^ab^	6.25 ± 0.57^ac^	5.94 ± 0.48^ac^
LVDD (mm)	4.04 ± 0.38	6.87 ± 0.78^a^	5.25 ± 0.43^ab^	6.93 ± 0.69^ac^	6.57 ± 0.57^ac^
LVEF (%)	70.11 ± 8.91	40.14 ± 3.78^a^	57.89 ± 3.56^ab^	38.23 ± 4.12^ac^	43.67 ± 4.54^ac^
IS (%)	0	47.48 ± 3.48^a^	34.35 ± 5.18^ab^	55.28 ± 4.71^ac^	50.38 ± 3.49^ac^
CVF (%)	1.30 ± 0.38	37.48 ± 4.12^a^	15.15 ± 1.71^ab^	34.35 ± 3.97^ac^	27.43 ± 3.10^ac^

*I/R, ischemia/reperfusion; HMGB1, high mobility group box 1 protein; LY, LY294002; SOD, superoxide dismutase; MDA, malondialdehyde; LVIDs, left ventricular systolic diameter; LVIDd, left ventricular end-diastolic diameter; LVEF, left ventricular ejection fraction; IS, infarction size; CVF, collagen volume fraction. “^*a*^” vs. Sham: *P* < 0.05; “^*b*^” vs. I/R: *P* < 0.05; “^*c*^” vs. HMGB1: *P* < 0.05.*

### Infarct Size

**FIGURE 1:**
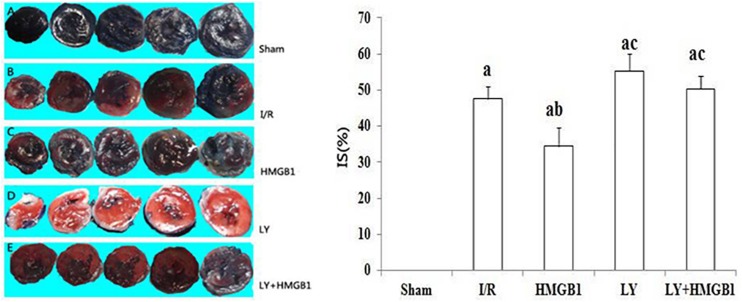
Infarct size in each groups. I/R, ischemia/reperfusion; HMGB1, high mobility group box 1 protein. LY, LY294002; IS, infarction size; IS (%) the percentage of the total section area of left ventricle occupied by the infarct. “a” vs. Sham: *P* < 0.01; “b” vs. I/R: *P* < 0.05; “c” vs. HMGB1: *P* < 0.01.

The average IS in the HMGB group was significantly smaller than in the I/R group (*P* < 0.05) (Figure 1). However, both the LY and the LY + HMGB1 groups had larger IS than the rats treated with HMGB1 (*P* < 0.01 for both), suggesting that LY294002 abolished the protective effects of HMGB1 against I/R-induced myocardial damage.

### Expression of VEGF Protein

**FIGURE 2:**
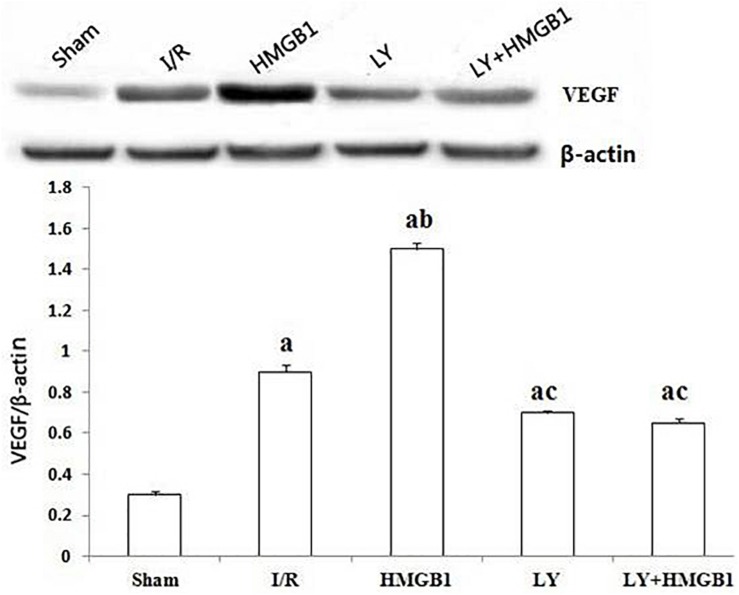
VEGF protein expression in each groups. I/R, ischemia/reperfusion; HMGB1, high mobility group box 1 protein; LY, LY294002; “a” vs. Sham: *P* < 0.01; “b” vs. I/R: *P* < 0.01; “c” vs. HMGB1: *P* < 0.01.

As demonstrated in Figure 2, in comparison with the sham-operated rats, the expression of VEGF protein was significantly increased in the I/R group (*P* < 0.01), and the treatment with HMGB1 further upregulated this growth factor (*P* < 0.01 vs. sham and I/R). However, both the LY and the LY + HMGB1 groups showed significantly lower VEGF expression than the HMGB1 group (*P* < 0.01 for both).

### Tissue Fibrosis

At 4 weeks after the surgery, CVF in the HMGB1-treated animals was significantly lower than in the I/R group (*P* < 0.05). Moreover, CVF in the LY and LY + HMGB1 groups was markedly higher than in the HMGB1 group (*P* < 0.05).

### Phosphorylation of Akt Protein

**FIGURE 3:**
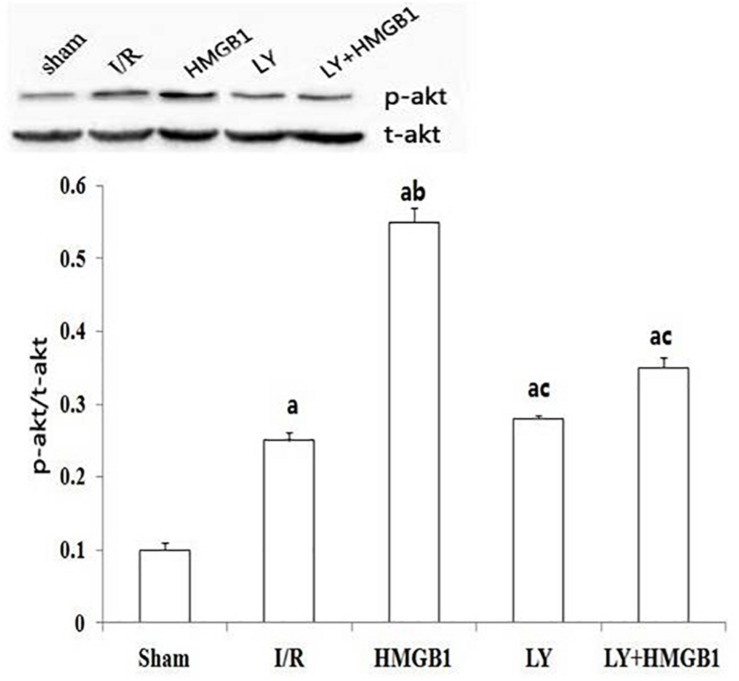
p-Akt expression in each groups. I/R, ischemia/reperfusion; HMGB1, high mobility group box 1 protein; LY, LY294002. “a” vs. Sham: *P* < 0.01; “b” vs. I/R: *P* < 0.01; “c” vs. HMGB1: *P* < 0.01.

The level of p-Akt protein was significantly higher in the I/R group than in the sham group (*P* < 0.01), and the treatment with HMGB1 further increased the phosphorylation of Akt (*P* < 0.01 vs. I/R) (Figure 3). However, the level of p-Akt protein in the LY and LY + HMGB groups was significantly lower than in the HMGB1-treated rats (*P* < 0.01 for both).

### Cardiac Function

As shown in Table 1, I/R injury resulted in a significant decrease in LVEF and LVFS and a significant increase of LVEDD at 44 weeks after the surgery (*P* < 0.01 vs. sham for all variables). In comparison with the I/R group, LVEF and LVFS in the HMGB1-treated rats were markedly increased, while LVEDD was significant (*P* < 0.05 for all variables). However, LY294002 reversed the protective effects of HMGB1 in the LY and LY + HMGB1 groups.

## Discussion

The major findings of the current study are: (1) myocardial expression of VEGF was significantly increased in I/R rats; (2) intravenous injection of HMGB1 resulted in significant protection from oxidative stress, reduced IS, increased myocardial expression of VEGF, attenuated myocardial fibrosis, and improved cardiac function in the rat model of I/R injury; (3) the cardioprotective effects of intravenous HMGB1 may be associated with the upregulation of VEGF protein expression; and (4) treatment with LY294002, a PI3K inhibitor, blocked the phosphorylation of Akt protein and abolished the cardioprotective effects of intravenously delivered HMGB1. Together, the obtained results suggest that intravenous administration of HMGB1 may ameliorate myocardial I/R injury by upregulation of VEGF expression in the myocardium, via activation of the PI3K/Akt signaling pathway.

Acute MI continues to be the leading cause of mortality and morbidity worldwide. Increasing numbers of patients with acute MI are treated with percutaneous coronary interventions or thrombolysis to restore coronary blood flow to the affected segment of the myocardium and decrease IS. Paradoxically, reperfusion itself results in further myocardial injury, which negatively affects cardiac function and worsens the prognosis of acute MI patients. At present, no effective therapeutic strategies are available for the prevention of I/R injury.

It is well documented that excessive oxidative stress contributes to the pathogenesis of myocardial I/R injury. When the balance between the production of reactive oxygen species (ROS) and their scavenging is perturbed, irreversible damage to the cells occurs. ROS lead to oxidative damage to a variety of cellular components (Tullio et al., 2013). Myocardial I/R injury is characterized by excessive production of free radicals, which trigger oxidation, denaturation, crosslinking, and degradation of DNA, RNA, proteins, and polysaccharides, ultimately leading to cardiomyocyte apoptosis and necrosis (Zhang et al., 2016). Also, several other studies demonstrated that antioxidants or ROS scavengers decrease IS after myocardial I/R (Jiang et al., 2014).

The level of MDA reflects the extent of lipid peroxidation in various tissues, and SOD activity reflects the functional status of scavengers of oxygen free radicals. On this basis, MDA concentration is often used as an index of ROS formation, while SOD activity is utilized as an indicator of lipid superoxide levels in the ischemic myocardium (Yao et al., 2015). In agreement with these studies, we have found that the content of MDA increased and SOD activity decreased significantly in rats subjected to myocardial I/R. However, pretreatment with HMGB1 resulted in the decrease of MDA content and reduction in IS while markedly increasing SOD activity I/R rats. As an unsaturated fatty acid, MDA contributes to the process of myocardial I/R injury and reflects the overall degree of lipid peroxidation. SOD can scavenge oxygen free radicals, thus protecting the heart from I/R injury (Tsutsui et al., 2011; Yao et al., 2016). On this basis, the current results provide further evidence that the cardioprotective effects of intravenously delivered HMGB1 resulting in the attenuation of myocardial I/R injury may be mediated by maintaining the balance between intracellular oxidants and antioxidants (Vikram et al., 2017).

Oxidative stress causes myocardial injury and cardiomyocyte death through various mechanisms. However, whether the reduction in the level of ROS can decrease IS remains controversial. Some studies have claimed the lack of beneficial effect of antioxidants and free radical scavenges on the size of an infarct generated by cardiac I/R (Nejima et al., 1989; Vanhaecke et al., 1991). Further research on the role of ROS in triggering myocardial damage is necessary to elucidate the mechanism of the protective effect of HMGB1 in myocardial I/R.

Vascular endothelial growth factor has a critical function in myocardial angiogenesis and vascular leakage. The expression of VEGF is significantly upregulated in chronically ischemic myocardium (Banai et al., 1994). Its levels increase rapidly after occlusion of coronary artery (Hashimoto et al., 1994), and enhanced expression of VEGF protects the heart from I/R injury (Zhou Y. et al., 2017). VEGF improves the proliferation and migration of vascular endothelial cells, promotes angiogenesis, inhibits cardiomyocyte apoptosis, and induces the growth of collateral blood vessels (Chen et al., 2016). Seo et al. (2017) demonstrated that increased secretion of VEGF potentiates therapeutic efficacy of human mesenchymal stem cells in the heart after I/R. We have previously demonstrated that myocardial expression of the VEGF mRNA in a rat model of myocardial I/R was significantly higher than in sham-operated animals. Importantly, the expression of VEGF mRNA was positively correlated with the improvement of cardiac function (Yao et al., 2013a).

Vascular endothelial growth factor has a beneficial function in several other tissues. In the kidneys, the release of VEGF promotes cell proliferation and angiogenesis, resulting in protection during acute renal I/R injury (Xue et al., 2017). VEGF is also an important prosurvival factor capable of decreasing neuronal death and improving neurological function (Khan et al., 2015). In agreement with this notion, inhibition of VEGF expression results in decreased angiogenesis after cerebral infarction, aggravating the injury of the brain tissue (Liang et al., 2016). Moreover, elevated circulating VEGF protects the aging liver against I/R injury (Limani et al., 2016).

Despite the available information on multiple functions of VEGF, it should be recognized that the role of this growth factor in the cardioprotective effects of intravenously administered HMGB1 is unclear. In the present study, the myocardial level of VEGF was higher in the I/R than in the sham group, suggesting that myocardial ischemia and hypoxia can upregulate VEGF expression. Pretreatment with HMGB1 prior to I/R resulted in a further increase in the myocardial expression of VEGF, indicating that the cardioprotective effect of HMGB1 may be mediated by the enhancement of VEGF expression. However, data suggesting the opposite were obtained by Aaron and colleagues who reported that intracoronary delivery of HMGB1 improved cardiac function after global I/R was associated with VEGF levels (Abarbanell et al., 2011). This apparent discrepancy might be due to the different animal models and the route and time window of drug administration used in these two studies. Thus, additional studies are warranted to identify the reason for the contrasting results and clarify the mechanism underlying the impact of VEGF on the outcome of myocardial I/R.

Oxidative stress increases the production of inflammatory cytokines, such as interleukin-1β and tumor necrosis factor-α. These cytokines trigger the inflammatory cascade, activating matrix metalloproteinases and deposition of collagen, ultimately resulting in myocardial fibrosis (Zornoff et al., 2009; Zhou et al., 2014). Excessive interstitial fibrosis leads to the stiffening of the ventricular wall and septum, progressive worsening of cardiac function, and increased morbidity and mortality. Ventricular remodeling is a continuous pathological process that results in the deterioration of cardiac anatomy and function after the primary ischemic insult. The development of new therapeutic strategies to control cardiac fibrosis and adverse ventricular remodeling is a subject of a significant research effort aiming at the prevention of cardiac dysfunction following acute MI (Sutton and Sharpe, 2000; Tallquist and Molkentin, 2017).

The current work documented that I/R significantly increases the level of VEGF in the myocardium, and HMGB1 further enhances VEGF expression, attenuating the myocardial injury caused by I/R. Additionally, intravenously administered HMGB1 counteracted the increase in CVF and the decrease in LVEF and LVFS induced by I/R injury. These results suggest that decreased IS ameliorated myocardial fibrosis, and improved cardiac function may be dependent on the changes in VEGF expression. However, the signaling pathway mediating the HMGB1-induced upregulation of VEGF after I/R is yet to be identified. Accumulating evidence points to the PI3K/Akt signaling pathway as the key mediator in the protection of the heart against the I/R injury (Chen et al., 2014; Yu et al., 2014).

The Akt protein plays an important role in the control of VEGF expression by increasing the protein level of HIF-1α, the upstream regulator of VEGF that promotes VEGF release (Ackah et al., 2005). Our laboratory has previously demonstrated that HMGB1 administered intravenously can increase the expression of HIF-1α in the myocardium via the PI3K/Akt signaling pathway (Yao et al., 2016). However, these findings do not answer the question of whether the PI3K/Akt signaling pathway is involved in the upregulation of VEGF expression by intravenous HMGB1. The current study has shown that HMGB1 can enhance the expression of VEGF, decrease LVID, LVDD, and IS, and increase LVEF in I/R rats. The treatment with LY294002, an inhibitor of PI3K, resulted in a significant increase in LVID, LVDD, and IS and a marked decrease in LVEF and VEGF expression. Also, intravenously delivered HMGB1 increased the phosphorylation of Akt. These results suggest that HMGB1 may result in the upregulation of VEGF, amelioration of myocardial I/R injury, and improvement of cardiac function by activating PI3K/Akt signaling pathway. LY294002 abolished the HMGB1-induced myocardial expression of VEGF and improvement of heart function, essentially eliminating the cardioprotective effects of intravenous HMGB1. Thus, the hypothesis can be advanced that HMGB1 enhances the myocardial expression of VEGF, and this effect is dependent, at least in part, on Akt phosphorylation. Additionally, the possibility is raised that the PI3K/Akt signaling pathway may be involved in the cardioprotective effects, including the recovery of cardiac function, afforded by HMGB1 delivered intravenously prior to myocardial I/R injury.

We have previously demonstrated that up-regulation of HIF-1α and VEGF by bFGF may exert a protective effect on the myocardium after I/R injury, and the upregulation of HIF-1α by HMGB1 is mediated by the activation of the PI3K/Akt pathway (Yao et al., 2016). A recent study has shown that exogenous HMGB1 significantly upregulated the expression of VEGF at both mRNA and protein levels by triggering phosphorylation of Akt, and inhibition of PI3K markedly suppressed VEGF overproduction in ARPE-19 cells (Chang et al., 2017). HMGB1 is a cytokine mediator localized in the nuclei of essentially all eukaryotic cells (Yang et al., 2013; Andersson et al., 2018). HMGB1 promotes inflammatory response (Hou et al., 2018) functioning as an essential facilitator in inflammatory diseases and has an important role in the pathophysiology of atherosclerosis (Kigerl et al., 2018). As a strong chemoattractant and a signal of tissue damage, HMGB1 promotes proliferation, migration, and differentiation of several types of stem cell, preventing left ventricular remodeling and enhancing left ventricular function after MI (Palumbo et al., 2004; Limana et al., 2005). Administration of HMGB1 contributes to the upregulation of VEGF, neovascularization of the infracted region of the heart, and improvement of cardiac function (Jiang et al., 2016). In agreement with these studies, the present investigation determined that exogenous HMGB1 can upregulate the expression of VEGF, and inhibition of PI3K/Akt signaling can abolish the protective effect of HMGB1 in the post-I/R myocardium. Furthermore, given that VEGF is a downstream factor of HIF-1α, we concluded that exogenous HMGB1 could play a protective role in I/R myocardium by upregulating the expression of VEGF through the PI3K/Akt signaling pathway (Yao et al., 2016).

Although additional mechanisms may be involved, the current investigation provides novel evidence that the cardioprotective effects of HMGB1 after I/R injury are, at least in part, regulated by the PI3K/Akt pathway. The underlying mechanisms may involve inhibition of oxidative stress, increased myocardial expression of VEGF, and attenuation of myocardial fibrosis. Together, these effects result in a decrease in IS and an improvement in cardiac performance.

Some limitations of the study have to be acknowledged. (1) The injection of HMGB1 was performed 30 min before the induction of ischemia. This protocol does not reflect the clinical reality because most MI patients are admitted to the hospital several hours after the event. Thus, studies of the effect of administration of HMGB1 at 3–4 h after myocardial I/R injury would be more clinically relevant. (2) Cardiac fibroblasts are important structural components of the heart and contribute significantly to ventricular remodeling and fibrosis following ischemic injury (Tallquist and Molkentin, 2017). Despite their relevance, the changes of fibroblasts were not assessed in this study to document their role in the effects of HMGB1 on the expression of VEGF and cardiac function in the rat model of myocardial I/R injury. (3) Genetically engineered models, e.g., VEGF knockdown, were not used to prove the mechanisms involved in the improvement of myocardial fibrosis and cardiac function by HMGB1. (4) Only the role of PI3K/Akt signaling pathway in myocardial fibrosis was addressed, and other myocardial signaling pathways were not analyzed. Thus, further studies are necessary to expand the understanding of the mechanisms of beneficial effects of HMGB1.

## Conclusion

In conclusion, we have demonstrated that the cardioprotective and heart function-improving effects of intravenously delivered HMGB1 are mediated by the enhancement of VEGF expression in the myocardium through the activation of PI3K/Akt signaling pathway. In view of the accumulated results, the intravenous administration of HMGB1 appears as a promising therapeutic strategy for treating myocardial I/R injury. However, further studies are required to determine the exact underlying molecular mechanism by which HMGB1 exerts its cardioprotective effects.

## Data Availability Statement

The raw data supporting the conclusions of this article will be made available by the authors, without undue reservation, to any qualified researcher.

## Ethics Statement

The animal study was reviewed and approved by the Ethics Committee of Liaocheng People’s Hospital, Liaocheng, China.

## Author Contributions

Y-HZ, Q-FH, LG, YS, Z-WT, MW, and WW designed and performed all the experimental work. H-CY and LG designed the research. H-CY and Y-HZ wrote the manuscript. H-CY, Y-HZ, and LG reviewed and edited the manuscript. All authors read and approved the manuscript to be published.

## Conflict of Interest

The authors declare that the research was conducted in the absence of any commercial or financial relationships that could be construed as a potential conflict of interest.
